# Impedance Technique-Based Label-Free Electrochemical Aptasensor for Thrombin Using Single-Walled Carbon Nanotubes-Casted Screen-Printed Carbon Electrode

**DOI:** 10.3390/s22072699

**Published:** 2022-03-31

**Authors:** Kyungsoon Park

**Affiliations:** Department of Chemistry and Cosmetics, Jeju National University, Jeju 63243, Korea; kspark895@jejunu.ac.kr; Tel.: +82-64-754-3545

**Keywords:** aptasensor, screen-printed carbon electrode (SPCE), thrombin-binding aptamer (TBA), electrochemical impedance spectroscopy (EIS), label-free biosensor

## Abstract

An impedance technique-based aptasensor for the detection of thrombin was developed using a single-walled carbon nanotube (SWCNT)-modified screen-printed carbon electrode (SPCE). In this work, a thrombin-binding aptamer (TBA) as probe was used for the determination of thrombin, and that was immobilized on SWCNT through π–π interaction. In the presence of thrombin, the TBA on SWCNT binds with target thrombin, and the amount of TBA on the SWCNT surface decreases. The detachment of TBA from SWCNT will be affected by the concentration of thrombin and the remaining TBA on the SWCNT surface can be monitored by electrochemical methods. The TBA-modified SWCNT/SPCE sensing layer was characterized by cyclic voltammetry (CV). For the measurement of thrombin, the change in charge-transfer resistance (*R_ct_*) of the sensing interface was investigated using electrochemical impedance spectroscopy (EIS) with a target thrombin and [Fe(CN)_6_]^3−^ as redox maker. Upon incubation with thrombin, a decrease of *R_ct_* change was observed due to the decrease in the repulsive interaction between the redox marker and the electrode surface without any label. A plot of *R_ct_* changes vs. the logarithm of thrombin concentration provides the linear detection ranges from 0.1 nM to 1 µM, with a ~0.02 nM detection limit.

## 1. Introduction

Aptamers are single-stranded DNA or RNA (ssDNA or ssRNA) molecules that can bind to a variety of specific targets, such as peptides [1,2], proteins [3,4], small molecules [5], viruses [6], and even live cells [7]. Compared with the conventional antibodies, the application of aptamer in bioassay has tremendous advantages, such as small-size, high reproducibility, easy modification, and better chemical stability [8,9]. Among the various sensing methods, electrochemical analysis is the most widely used one in the field of aptasensor due to their high sensitivity, low cost, low detection limit and fast response time [10,11,12]. The electrochemical detection method typically requires immobilization of probe molecules on a solid substrate to form a sensing layer while maintaining the activity of the immobilized biomolecules. The most widely employed immobilization strategy is self-assembled monolayers (SAMs), which is generally used for surface modification of biosensors [13,14,15]. Organic SAMs can provide a suitable interface layer for developing biosensors which are stable, uniform, and have terminal functional groups that can be modified easily [16]. However, for electrochemical biosensors, SAMs can cause an electron transfer hindrance between the electrode and redox maker [17]. In this regard, carbon nanotubes (CNTs) can be used to overcome this insulating issue. CNTs have good electrical conductivity, mechanical properties [18], and catalytic effects [19,20,21], so they can provide a conducting matrix for electrochemical detection by the biosensors [22,23,24]. Specially, aptamers like ssDNA can bind to SWCNT through π–π interaction without any chemical modification or reaction [25,26,27,28]. Therefore, SWCNT serving as conducting support can be a convenient and strong platform for electrochemical aptasensor.

Screen-printed carbon electrode (SPCE)-based disposable sensor has been of great interest in bioassay applications for point-of-care and early diagnostics. The SPCE is a useful analytical tool that can meet the demands for portable, low-cost, miniaturization and reduction of analyte volume in electrochemical analysis [29,30,31]. Also, electrochemical impedance spectroscopy (EIS) is another powerful and useful tool to measure interfacial electrical properties which can be used to monitor bio-recognition events occurring at the electrode surface [32,33,34]. EIS is a non-destructive technique where a small amplitude perturbation allows direct detection of the target molecules from measured impedance changes of the sensing layer without any label [35,36].

Thrombin, a serine protease, is known to regulate platelet aggregation and modulate various coagulation-related metabolism in humans. Also, thrombin is not only related to hemostasis or thrombosis disorder, but also to some fatal diseases, such as Alzheimer’s disease, Parkinson’s disease, atherosclerosis, and tumor growth [37,38]. Therefore, rapid control of thrombin concentration is crucial for the prevention, control, and regulation of thrombin-associated diseases. 

In the present report, we have introduced a label-free impedance technique-based aptasensor to sense thrombin using a single-walled carbon nanotube (SWCNT)-casted screen-printed carbon electrode (SPCE). The SWCNT-casted SPCE was used as a sensing platform for this system. The thrombin binding aptamer (TBA), as a probe for thrombin, binds to the SWCNT-casted SPCE by π–π stacking. In the presence of thrombin, the TBA becomes detached from the SWCNT to bind with the thrombin. This selective bio-recognition events can be monitored by EIS, which relies on the difference in the charge-transfer resistance (*R_ct_*) between negatively charged Fe(CN)_6_^3−/4−^_,_ a redox maker and remaining TBA, which is also negatively charged. The EIS spectra in the form of Nyquist plots were analyzed using a Randles circuit, and *R_ct_* decrease was found to be proportional to the thrombin concentration. The detection limit of the developed thrombin sensor was ~0.02 nM, and the detection range was from 0.1 nM to 1 uM of thrombin concentration.

## 2. Materials and Methods

### 2.1. Reagents

Goat anti-mouse IgG was purchased from EMD Millipore (Burlington, MA, USA). The disposable SPCE were purchased from DropSens (Asturias, Spain, ref. 150). Unless otherwise mentioned, all reagents and chemicals were obtained from Sigma-Aldrich (St. Louis, MO, USA) and used as received. The TBA oligonucleotides (HPLC purified) were synthesized from Geno-Tech (Daejeon, Korea) and the sequence of TBA used was 5′-AGTCCGTGGTAGGGCAGGTTGGGGTGACT-3′ (29-mer). All the chemicals and reagents used in this work were of reagent grade, and ultrapure water (>18 MΩcm) was obtained from a Millipore Milli-Q purification system.

### 2.2. Preparation of TBA-SWCNT/SPCE Sensing Platform

Electrochemical pretreatment procedure was applied to SPCE prior to their use. Pretreatment of SPCE is an important step for the enhanced electrochemical properties (e.g., electron transfer kinetics, impedance, background current, and voltametric signal). The electroactive surface area (0.0858 cm^2^) of SPCE was calculated using the Randle–Sevcik equation for quasi-reversible process (Figure S1). In this procedure, a potential (ranging from −1.2 to 1.2 V vs. Ag QRE) was applied for 15 cycles in 0.1 M H_2_SO_4_, followed by deionized water washing and N_2_ drying. 0.50 mg of SWCNT was dispersed in 1 mL mixture of sulfuric and nitric acid (3:1) with the aid of ultrasonication to obtain a homogeneous and well-dispersed suspension. Next, 10 µL of this suspension were dropped onto the pretreated working electrode of SPCE and solvent was evaporated in air. The attachment of 100 µM TBA as probe (Figure S1) to SWCNT-modified SPCE was carried out by incubation in 10 µL of incubation buffer (IB; 50 mM Tris-HCl, 5 mM KCl and 100 mM NaCl, pH 7.4) containing TBA for 30 min (Figure S2). Those SPCE were washed with DNA rinsing buffer (DNA-RB; 5 mM Tris-HCl and 10 mM NaCl, pH 7.4) and dried with N_2_ gas. The SPCE/SWCNT-TBA electrode was exposed to 100 µL of various concentrations of the thrombin as target molecules for 30 min. After incubation with the target protein, the electrode was washed with protein rinsing buffer (PRO-RB; 50 mM Tris-HCl, 0.5 M NaCl and 0.05% (*w*/*w*) Tween 20, pH 7.4) and dried with N_2_ gas.

### 2.3. AFM and Electrochemical Measurement

**Figure 1:**
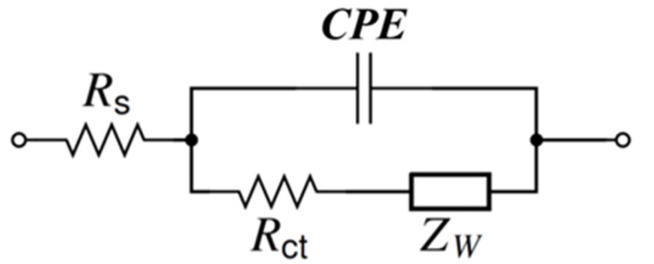
Representation of Randles equivalent circuit for the data fitting.

The surface analysis of the SPCE was obtained with a NX10 AFM system (Park Systems, Suwon, Korea), using a PPP-NCHR cantilever (Nanosensors). Measurements were recorded under tapping mode of AFM. The surface morphology of bare SPCE and SWCNT-modified SPCE were characterized by field-emission scanning electron microscopy (FE-SEM, TESCAN, MIRA III). Cyclic voltammetry was performed using a CHI6011e potentiostat (CH Instrument, Bee Cave, TX, USA). For EIS measurement with frequency response analyzer (FRA), GAMRY interface 1010e potensiostat was used. SPCE consisted of a carbon working electrode (4 mm diameter), a silver pseudo-reference electrode and a platinum counter electrode deposited on a ceramic support. For cyclic voltammetry, the electrolyte is the aqueous solution with 1.0 mM ferricyanide and 0.1 M KNO_3_ as supporting electrolyte. EIS measurements were obtained in 1.0 mM Fe(CN)_6_^3−^/1.0 mM Fe(CN)_6_^4−^ (1:1 molar ratio) and 0.1 M KNO_3_ in electrochemical buffer (EB; 0.1 M phosphate buffer and 700 mM NaCl, pH 7.4). The frequency ranges were scanned from 100 kHz to 0.2 Hz with an AC magnitude of 10 mV rms at 0.190 mV versus Ag QRE as a DC bias potential. The impedance spectra plotted in the complex plane (Nyquist plots, Z″ vs. Z′) and fitted to a Randles equivalent circuit (Figure 1), which include the solution resistance (*R_s_*), the charge-transfer resistance (*R_ct_*), a constant phase element (*CPE*) and the Warburg impedance (*Z_w_*) using GAMRY software.

## 3. Results

### 3.1. Characterization of TBA-Modified SWCNT/SPCE

**Scheme 1:**
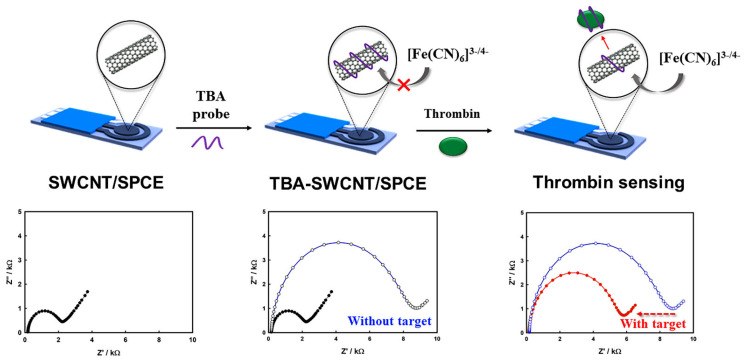
Schematic illustration of a label−free aptasensor for thrombin detection using a SWCNT casted SPCE. Inset: EIS spectra with (red) and without (blue) target thrombin on TBA−SWCNT/SPCE. Nyquist plot of SWCNT/SPCE (black) was represented.

**Figure 2:**
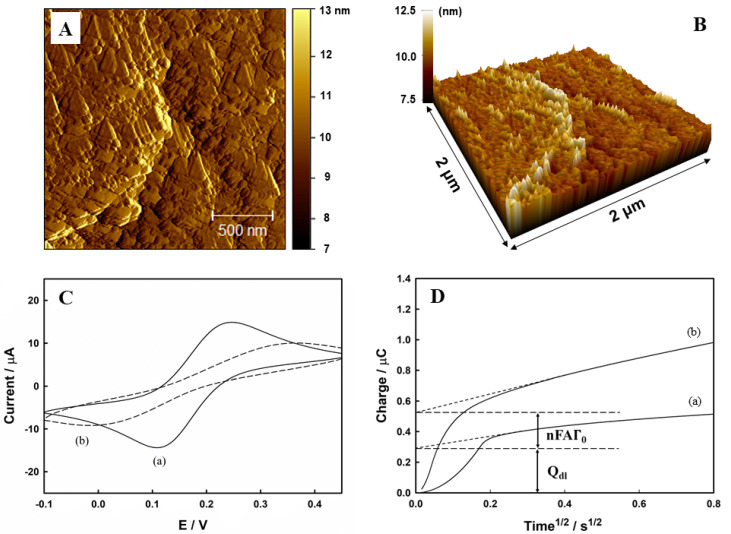
(**A**) 2D and (**B**) 3D AFM images of bare SPCE. (**C**) Cyclic voltammograms of (a) SWCNT−casted SPCE (SWCNT/SPCE) and (b) after being incubated with 50 µM TBA in a EB with [Fe(CN)_6_]^3−/4−^ and 0.1 M KCl. The scan rate was 0.1 V/s. (**D**) Chronocoulometric response plots for TBA−modified SWCNT/SPCE in the (a) absence and (b) presence of 50 µM Ru(NH_3_)_6_^3+^. The dashed line represents the fit to the experimental data used to obtained the intercept charges at *t* = 0.

The SWCNTs were casted on SPCE and incubated in a solution containing TBA, as shown in Scheme 1. The 2D and 3D AFM images of the bare SPCE surface were presented in Figure 2A,B, respectively. The AFM image showed that the bare SPCE had a rough surface interspersed with triangular and circular groupings of various sizes. The RMS roughness value (*Rq*) obtained for a scanned area of 2 × 2 µm^2^ of the bare SPCE surface was ~0.881 nm. The field-emission scanning electron microscopy (FE-SEM) measurements were performed to image the deposition of SWCNT on SPCE (Figure S4). Also, to verify the immobilization of the TBA onto the SWCNT, cyclic voltammetry was run using a Fe(CN)_6_^3−^ as redox maker.

As shown in Figure 2C(a), the SWCNT-modified SPCE (SWCNT/SPCE) showed a characteristic quasi-reversible redox cycle with peak-to-peak separation of ca. 130 mV. These results proved that the SWCNT/SPCE can act as a suitable sensing support without interfering electron transfer. Compared with the redox behavior of SWCNT/SPCE, the TBA-modified electrode (TBA-SWCNT/SPCE) showed a slower charge kinetics, as shown in Figure 2C(b). These results are in good agreement with the conventional electrochemical behavior of a DNA-modified electrode reported previously [39]. Upon assembly of the TBA onto the SWCNT/SPCE, the peak-to-peak separation increased remarkably without any change in the formal potential (E° = (E_pa_ + E_pc_)/2), while the peak current decreased due to repulsion between the negatively charged redox probe ions and the polyanionic TBA. 

Furthermore, surface density of the TBA on SWCNT/SPCE was calculated from the integrated Cottrell equation using Chronocoulometry (CC) [40]. CC is a current integration method which measures the total charge flow under equilibrium conditions. The CC intercept of the charge vs. *t*^1/2^ plot at *t* = 0 indicates the total charge of double layer capacitance and the surface-confined molecule. The conventional chronocoulometric response with Ru(NH_3_)_6_^3+^ at TBA-SWCNT/SPCE was obtained (see Figure 2D). The surface-adsorbed Ru(NH_3_)_6_^3+^ at a TBA was determined from the difference between the CC intercepts for the absence or presence of Ru(NH_3_)_6_^3+^. The surface coverage of Ru(NH_3_)_6_^3+^ was then converted to the surface density of TBA on SWCNT/SPCE using Equation (1):

(1) $$\Gamma_{TBA}=\Gamma_{0}(z/m)$$

where Γ*_TBA_* is the surface density of TBA in mol/cm^2^, Γ_0_ is surface-adsorbed Ru(NH_3_)_6_^3+^ in mol/cm^2^, *z* is the charge of the redox molecule and *m* is the number of bases in the TBA. A surface density of 2.56 × 10^−12^ mol/cm^2^ TBA on SWCNT/SPCE was estimated using Equation (1). From these results, we can confirm the immobilization of TBA on SWCNT/SPCE and the ability of sensing layer for further target detection.

### 3.2. TBA-SWCNT/SPCE with Target Molecule

The binding enthalpy between TBA and SWCNT differs depending upon the DNA sequence; however, in our system, it can be estimated as several thousand kJ/mol (−1.17 eV/nm for poly(T) strands) [25,41]. On the other hand, the dissociation constant (*K_d_*) of 29 mer TBA for thrombin was reported as 0.5 nM, which corresponded to 53 kJ/mol of free energy [42,43]. Depending upon the dissociation constant values, the TBA interacts more strongly with SWCNT than with thrombin. Therefore, the SWCNT-modified TBA was less removed by low concentration of the target thrombin. Despite the low binding affinity between the TBA and thrombin, the binding probability can be affected not only by the binding affinity, but also by the concentration of the target molecule. Therefore, the detachment of TBA from SWCNT will be proportional to the concentration of the thrombin, and any remaining TBA on the SWCNT/SPCE surface can be determined by electrochemical method. 

**Figure 3:**
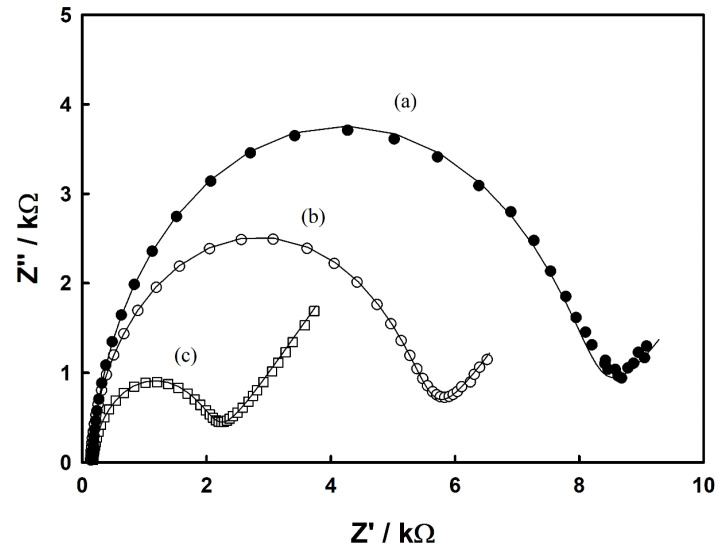
Impedance spectra in the presence of 0.1 mM Fe(CN)_6_^3−^/0.1 mM Fe(CN)_6_^4−^ (1:1 molar ratio) and 0.1 M KNO_3_ in EB using a different electrode (**a**) before and (**b**) after incubation with 100 nM thrombin as target protein on TBA−SWCNT/SPCE. (**c**) Nyquist plot of SWCNT/SPCE without TBA under the same electrolyte conditions. Solid curves represent the fitting results from an equivalent circuit.

EIS is a powerful tool for label-free sensing of target molecules. The electrochemical impedance was measured from the impedance between the sensor electrode and the electrolyte solution. The EIS experiments were performed to verify the sensing of the target protein by the TBA-modified SWCNT/SPCE. In this experiment, [Fe(CN)_6_]^3−/4−^ was used as electroactive marker ion. The interaction of charged redox species with charged probe layer can cause changes in charge-transfer resistance (*R_ct_*), which is related to the barrier properties of the electrode surface. To quantify the TBA−thrombin interaction, EIS was performed. On SWCNT/SPCE without TBA in Figure 3c, a small *R_ct_* (~1.9 kΩ) value was measured with a diffusion-controlled behavior called the Warburg impedance. After being incubated with thrombin, the *R_ct_* of the TBA-modified SWCNT/SPCE decreased in Figure 3b and became smaller than that in the absence of the thrombin in Figure 3a. Because of the partial removal of the TBA by target binding, the impedance data showed a smaller *R_ct_* due to decrease in the repulsive interaction between the redox marker ion and the electrode surface.

In the presence of thrombin, the binding between the TBA and thrombin causes a conformational change of TBA, which will interfere with the interaction between the TBA and SWCNT. As per this result, the TBA can be detached from the SWCNT surface, resulting in a decrease of negatively charged TBA. Consequently, this competitive interaction with the TBA to thrombin and SWCNT is a key role in this sensor system, which is applicable to our label-free faradic impedance detection.

### 3.3. Control Experiment

**Figure 4:**
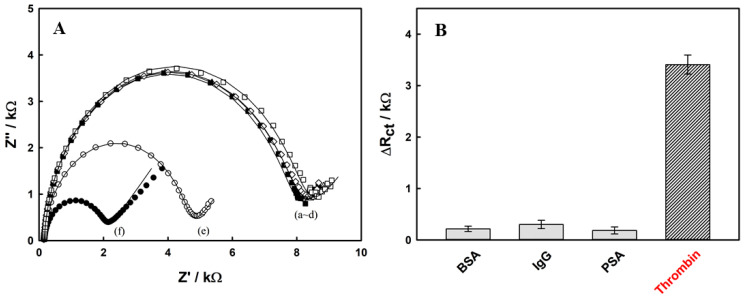
(**A**) Impedance spectra at TBA-modified SWCNT/SPCE (a) before and after (b−e) incubation with protein: (b) 150 nM PSA, (c) 120 nM IgG, (d) 200 nM BSA, or (e) 100 nM thrombin in the presence of 0.1 mM Fe(CN)_6_^3−^/0.1 mM Fe(CN)_6_^4−^ (1:1 molar ratio) and 0.1 M KNO_3_ in EB. (f) Nyquist plot of SWCNT/SPCE without TBA under same electrolyte conditions. Solid curves represent the fitting results from an equivalent circuit. (**B**) Plot of the corresponding ∆*R_ct_*. ∆*R_ct_* = *R_ct,0_* − *R_ct,p_*. *R_ct,0_* is the *R_ct_* in the absence of protein and *R_ct,p_* is that in the presence of each protein. Errors bars are obtained based on three independent measurements.

To check the selectivity of the newly developed sensor system, EIS was obtained in the presence of non-target protein, such as bovine serum albumin (BSA), immunoglobulin G (IgG), and prostate-specific antigen (PSA), because these are the commonly used proteins in laboratory. The EIS result with target proteins (Figure 4A(e)) showed a clear decrease in the *R_ct_*. The control experiments with PSA (Figure 4A(b)), IgG (Figure 4A(c)) and BSA (Figure 4A(d)) showed a similar current level compared to the signal in the absence of target protein (Figure 4A(a)). The EIS signal with target protein only amounted to ~56% of the charge-transfer response obtained without any target protein.

As shown in Figure 4B, the ∆*R_ct_* response according to the target or non-target protein also clearly demonstrated that thrombin was specific to the TBA binding event, implying high selectivity of this sensor system. The selective detection of the target protein from various proteins is an important characteristic of the developed biosensor system. 

### 3.4. EIS Response According to the Target Concentration

**Figure 5:**
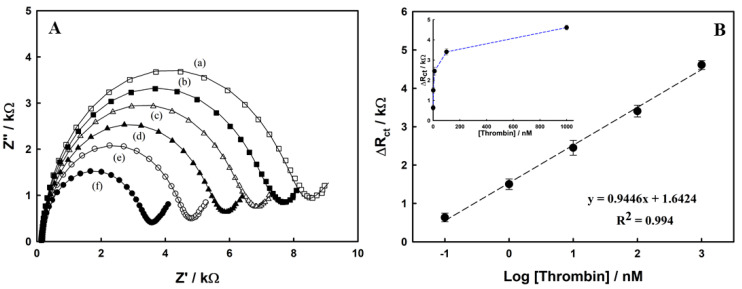
(**A**) Impedance spectra corresponding to various concentration of target protein: (a) 0 M, (b) 0.1 nM, (c) 1 nM, (d) 10 nM, (e) 100 nM and (f) 1.0 µM of thrombin. EIS experiments were obtained in EB solution containing 0.1 mM Fe(CN)_6_^3−^/0.1 mM Fe(CN)_6_^4−^ (1:1 molar ratio) and 0.1 M KNO_3_. Solid curves represent the fitting results from an equivalent circuit. (**B**) Linear calibration curve for ∆*R_ct_* vs. log (C_thrombin_/nM), where C_thrombin_ represents thrombin concentration. The inset of (**B**) is plots of ∆*R_ct_* against thrombin concentration. Errors bars are obtained based on three independent measurements.

The detection of thrombin at the TBA−modified SWCNT/SPCE was investigated with different concentrations of the target thrombin. Figure 5A shows Nyquist plots of TBA−SWCNT/SPCE in an EB solution containing 0.1 mM Fe(CN)_6_^3−^/0.1 mM Fe(CN)_6_^4−^ (1:1 molar ratio) and 0.1 M KNO_3_ after incubation with different concentrations of thrombin. In the absence of thrombin, a high-intensity charge−transfer barrier was observed in Figure 5A(a). Upon increasing the thrombin concentration from 0.1 nM to 1 µM, the SWCNT−confined aptamer underwent a bonding with the target molecule, which led to a decrease in the *R_ct_* between the redox maker ion and the electrode surface. The immobilization of TBA on the SWCNT/SPCE surface was crucial for this sensor system, because the amount of TBA on SWCNT directly affected the impedance signal. Figure 5A shows the sequential decrease in *R_ct_* from ~6.58 kΩ to ~3.21 kΩ (trace (b)~(f) in Figure 5A). The plot of the ∆*R_ct_* versus logarithm of thrombin concentration (Figure 5B) showed a linear relationship with ∆*R_ct_* = 0.945 logC + 1.6424 (*R*^2^ = 0.994), where *C* is the concentration of thrombin in nM. Using this plot, the detection limit (DL) of the aptasensor was calculated as ~0.02 nM based on DL = 3*S_b_*/*m* (*S_b_* is the standard deviation of the blank and *m* is the slope of the corresponding calibration curve). The concentration of thrombin in blood varies from nM to low µM levels. So, the sensitivity and detection range of our developed aptasensor is sufficient compared with various aptasensors for thrombin (see Table 1).

## 4. Conclusions

In this study, an electrochemical aptasensor with a TBA was developed using a SWCNT-casted SPCE for the detection of thrombin. The TBA-modified sensing layer was characterized using cyclic voltammetry (CV). The target protein and non-target protein can be discriminated selectively without any modification to the probe. The bio-recognition event between TBA and target thrombin was successfully investigated using impedance technique in 0.1 nM~1.0 µM detection ranges, with a ~0.02 nM detection limit. Additionally, the use of disposable SPCE can reduce the analyte volume and assay costs. This work demonstrated that the TBA-modified SWCNT/SPCE can be used as a sensitive and selective label-free EIS sensing flatform for the detection and quantification of thrombin.

## Figures and Tables

**Table 1:** Limit of detection (LOD) and range of various thrombin biosensors.

Detection Method/Label	LOD	Dynamic Range	Reference
Impedance/label-free	0.02 nM	0.1 nM~1.0 μM	This work
Fluorescent/label-free	0.03 nM	0.28 nM~86 nM	[44]
Electrochemiluminescence/Ru(bpy)32+	0.21 nM	0.5 nM~25 nM	[45]
Surface plasmon resonance/AuNPs	0.1 nM	0.1 nM~75 nM	[46]
Electrochemical/AuNPs and HRP	30 fM	0.1 pM~60 pM	[47]
Bioluminescent sensor/AuNPs	0.08 nM	8 nM~8 μM	[48]
Electrochemical/enzyme (GOD)	10 nM	10 nM~100 μM	[49]

## Data Availability

The data are publicly available.

## Supplementary Materials

The following supporting information can be downloaded at: https://www.mdpi.com/article/10.3390/s22072699/s1, Figure S1: (A) CVs of the bare SPCE depending on a scan rate in 0.5 mM Ru(NH_3_)_6_^3+^ and 0.1M KCl. (B) Plots of cathodic peak current according to the square root of the scan rate of (A). Figure S2: (A) The calibration plot of the charge transfer resistance (*R_ct_*) according to the concentration of TBA as probe molecules on SWCNT/SCPE for optimum conditions of this sensor system. (B) Plot of the corresponding *R_ct_* of (A) with error bars.; Figure S3: (A) The calibration plot of the ∆*R_ct_* according to the incubation time of thrombin as target molecules with TBASWCNT/SCPE for optimum conditions of this sensor system. (B) Plot of the corresponding ∆*R_ct_* of (A) with error bars. Figure S4: FE−SEM images of (A) bare SPCE (scale 200 nm), and (B) SWCNT modified SPCE (scale 200 nm). Reference [50] is cited in Supplementary Materials.
