# Supplementary material for: Impedance Technique-Based Label-Free Electrochemical Aptasensor for Thrombin Using Single-Walled Carbon Nanotubes-Casted Screen-Printed Carbon Electrode
Source: Sensors (Basel). 2022 Mar 31;22(7):2699. doi: 10.3390/s22072699 (PMC9002654; doi:10.3390/s22072699)
Supplement: Supplementary file 1 [file sensors-22-02699-s001.zip › sensors-1598231-supplementary.pdf]

Supplementary Material: Impedance Technique Based Label-free Electrochemical Aptasensor for Thrombin Using Single-walled Carbon Nanotubes Casted Screen-printed Carbon Electrode

Kyungsoon Park*

Department of Chemistry and Cosmetics, Jeju National University, Jeju 690-756, Korea

* Corresponding Authors: Prof. Kyungsoon Park

E-mail: kspark895@jejunu.ac.kr

Determination of the electroactive area of SPCE using Electrochemical method

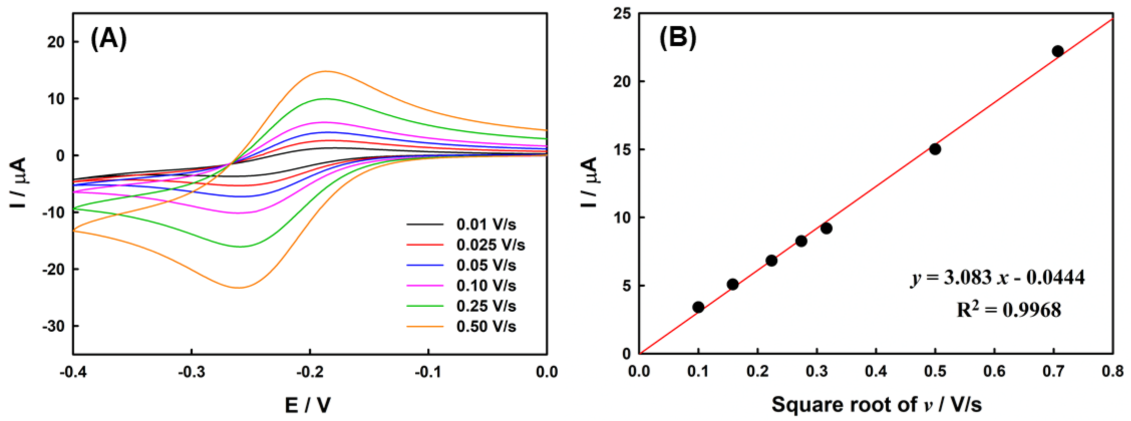

Figure S1. (A) CVs of the bare SPCE depending on a scan rate in 0.5 mM $Ru(NH_3)_6^{3+}$ and 0.1M KCl. (B) Plots of cathodic peak current according to the square root of the scan rate of (A).

The electroactive surface area of SPCE can be obtained using the Randle-Sevcik equation for quasi-reversible process at room temperature [50].

$$I_p = \pm 0.436\ nFA_{real}C\ (nFDv/RT)^{1/2}$$

Where I_p is the net peak current (A), n is the number of electron transferred in the redox event, A_{real} is the electroactive area of the electrode (cm^2), D is the diffusion coefficient of redox ($cm^2\ s^{-1}$), C is the bulk concentration of redox ($mol\ cm^{-3}$), F is the Faraday constant ($C\ mol^{-1}$), R is the universal gas constant, T is the temperature in Kelvin, and v is the scan rate ($V\ s^{-1}$). The calculated A_{real} and its percentage compared to the A_{geo} (%Real=(A_{real}/A_{geo}) × 100) is presented as follow:

A_{real} using Randle-Sevcik /cm^2	D/$cm^2\ s^{-1}$	% Real
0.0858	9.10×10^{-6}	68.3

Optimization of experimental conditions : Probe concentration

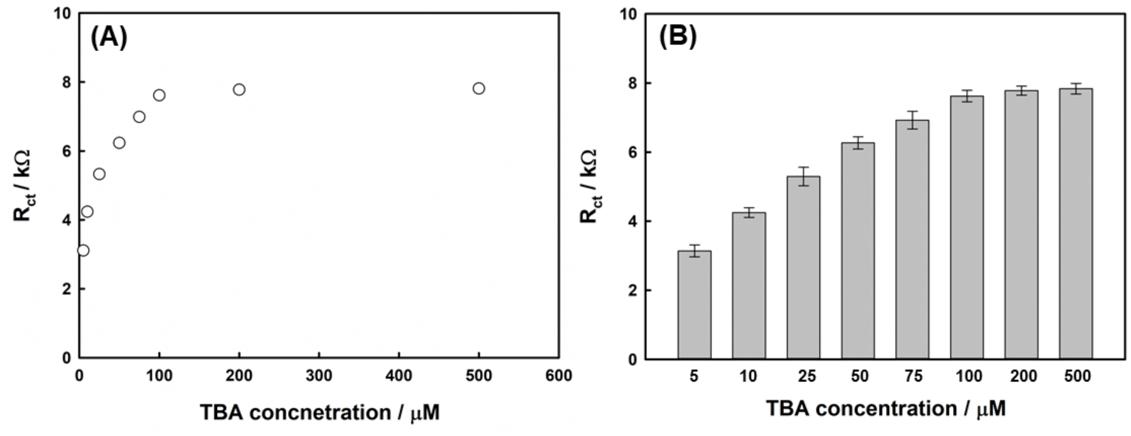

Figure S2. (A) The calibration plot of the charge transfer resistance (R_{ct}) according to the concentration of TBA as probe molecules on SWCNT/SCPE for optimum conditions of this sensor system. (B) Plot of the corresponding R_{ct} of (A) with error bars.

Optimization of experimental conditions : Target incubation time

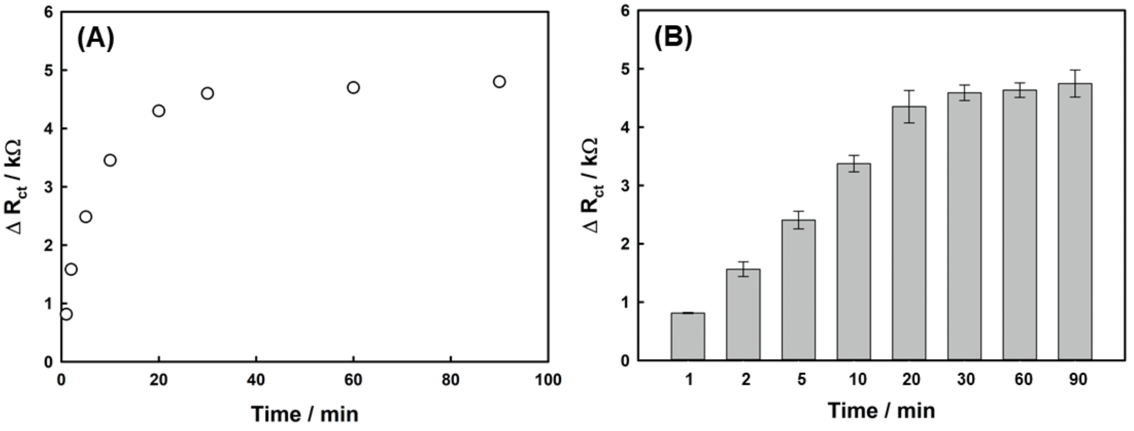

Figure S3. (A) The calibration plot of the ΔR_{ct} according to the incubation time of thrombin as target molecules with TBA−SWCNT/SCPE for optimum conditions of this sensor system. (B) Plot of the corresponding ΔR_{ct} of (A) with error bars.

FE-SEM images of bare SPCE and SWCNT modified SPCE

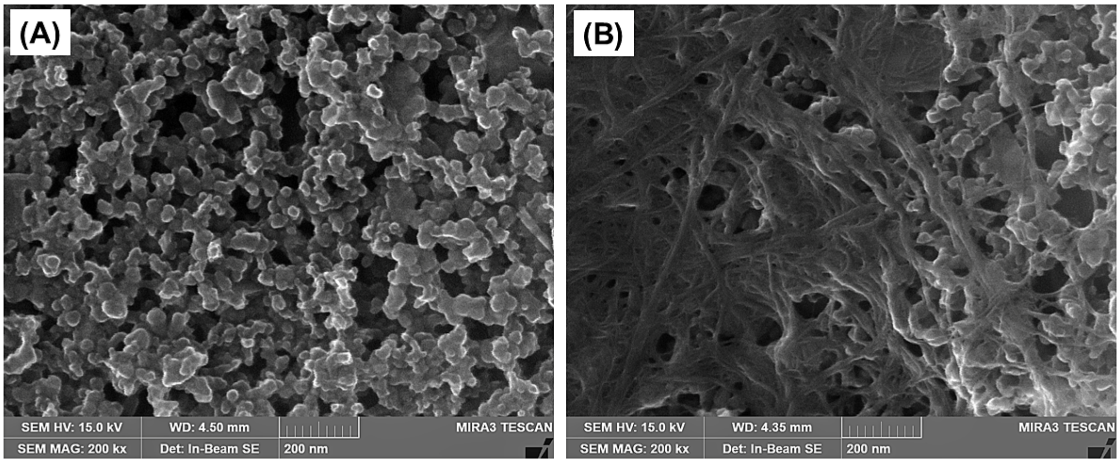

Figure S4. FE-SEM images of (A) bare SPCE (scale 200 nm), and (B) SWCNT modified SPCE (scale 200 nm).

To confirm the deposition of SWCNT on SPCE, the field-emission electron microscopy (FE-SEM) measurements were performed. As shown in Figure S4 (A), the graphitic carbon powder is dispersed throughout the bare SPCE and it showed the spherical shape with a wide particle size distribution. After modifying the SPCE with SWCNT (Figure S4 (B)), the presence of SWCNT which bridge and covered the bare SPCE and small particles of graphitic powder were observed.
